# Food allergy satisfaction with life scale: A complementary measure to health‐related quality of life

**DOI:** 10.1002/clt2.12233

**Published:** 2023-03-06

**Authors:** Gabriel Lins de Holanda Coelho, Melanie Lloyd, Mimi L. K. Tang, Audrey DunnGalvin

**Affiliations:** ^1^ School of Applied Psychology University College Cork Cork Ireland; ^2^ School of Public Health and Preventative Medicine Monash University Parkville Victoria Australia; ^3^ Allergy Immunology Group Murdoch Children's Research Institute Melbourne Victoria Australia; ^4^ Department of Allergy and Immunology The Royal Children's Hospital Melbourne Victoria Australia; ^5^ Department of Paediatrics The University of Melbourne Melbourne Victoria Australia

**Keywords:** food allergy, quality of life, satisfaction with life

To the Editor,

1

Food allergy (FA) significantly impacts patients and their families/caregivers. Allergen avoidance requires identifying and avoiding potential allergens and risky situations, managing allergic symptoms following accidental allergen exposure, and coping with the resulting psychosocial impacts of these burdens. The constant vigilance required to implement allergen avoidance and the ever‐present fear of unpredictable reactions has been shown to decrease health‐related quality of life (HRQL),^1^ as shown by the use of questionnaires like the FA quality of life questionnaire (FAQLQ)^2^ and FA anxiety scale (FAAS).^3^ However, such tools do not cover aspects such as how those impacted by FA cognitively evaluate their own lives. This evaluation is known as satisfaction with life (SwL).^4^ The construct is negatively linked to a range of distresses that can grow in a context of constant fear (as faced by many impacted by FA), such as depression, stress, and suicide ideation, and positively linked with indicators of a healthier life, such as optimism and self‐esteem.^5^

Here we introduce a FA‐specific SwL measurement tool. We adapted the well‐known satisfaction with life scale (SWLS)^4^ by directing its five items to focus on FA (For the full questionnaire, see Appendix). A disease‐specific questionnaire facilitates the measurement of the specific impact of the disease (i.e., FA) across a construct (i.e., SwL). A general questionnaire in studies of FA does not control for the influence of non‐FA‐related factors that might impact the construct, such as other daily events and burdens. On the other hand, disease‐specific questionnaires (e.g., FAQLQ,^2^ FAAS^3^) are more sensitive to the context in which they are being used, focusing on the disease. It is crucial to use measures that have been appropriately designed and tested in order to evaluate the HRQL accurately for individuals with FAs.^6^ Therefore, our adaptation can provide new perspectives in HRQL studies focused on FA.

We collected the data using Prolific (https://www.prolific.co/), a crowdsourcing platform with well‐known data quality.^7^ To participate in the study, potential participants had to satisfy the following pre‐screening criteria: being diagnosed with a FA, United Kingdom or Ireland as their birth country, having a minimum of 25 previous participations on Prolific, and having a perfect approval rate (i.e., never failing a study, 100% approval). Two hundred and one participants (128 women, 63.7%; 73 men, 36.3%; *M*_age_ = 37.12, SD_age_ = 0.482) answered the adapted food allergy satisfaction with life scale (FASWLS), the FAQLQ^2^ for adults, and the FAAS.^3^ Most participants were diagnosed by a general practitioner (*n* = 102, 50.7%) or allergist (*n* = 55, 27.4%). The most reported allergens were peanut (*n* = 79, 39.3%), tree nuts (*n* = 68, 33.8%), and shellfish (*n* = 38, 18.9%). A 148 (73.6%) reported having experienced a severe allergic reaction.

We performed the analysis using R Studio for Confirmatory Factor Analysis, and JASP (https://jasp‐stats.org/) to assess reliabilities and convergent validity. We performed Confirmatory Factor Analysis using the weighted least square mean and variance adjusted (WLSMV) estimator. We considered the following indices to assess model fit^8^: comparative fit index (CFI), Tucker–Lewis index (TLI), and Standardized Root Mean Squared Residual (SRMR). For CFI and TLI, recommended values are higher than 0.90, whereas SRMR should present values below 0.08. For the reliability levels (McDonald's omega and Cronbach's alpha), recommended values are over 0.70.^8^ Finally, to assess convergent validity, we performed Pearson's correlation.

We found a good model fit for the unidimensional structure of the FASWLS (CFI = 0.98, TLI = 0.96, SRMR = 0.027), with factorial loadings ranging from 0.60 (Item 05) to 0.90 (Item 02). The FASWLS had good reliability (Cronbach's alpha = 0.88, McDonald's omega = 0.89). It was also negatively correlated with the FA anxiety score (*r* = −0.51, *p* < 0.001), and positively with all FAQLQ factors: emotional impact (*r* = 0.52, *p* < 0.001), health (*r* = 0.47, *p* < 0.001), risk (*r* = 0.48, *p* < 0.001), and social and dietary limitations (*r* = 0.58, *p* < 0.001). In other words, participants who presented a higher score in satisfaction with FA life also presented lower scores in FA anxiety and higher scores in FA quality of life.

Our findings validate FASWLS as a psychometrically sound scale. The unidimensional structure presented a good model fit and reliability, with significant correlations to essential constructs. SwL is an essential psychological construct, and this adaptation can enhance HRQL research by providing a focused understanding of the link between FA and SwL.^4^ The FASWLS provides a standardized approach to assess a subjective aspect not covered by other currently available FA‐specific questionnaires, thereby capturing a broader and more comprehensive analysis of disease impact on the individual. Where quality of life questionnaires focus on objective aspects that can influence well‐being (such as participants' perceived risk in the FAQLQ), the FASWLS focuses on a cognitive evaluation of life overall, when living with FA.^4^ In practical terms, an individual could have a high FA HRQL due to a low perceived risk, but still have a low evaluation of their own life due to the simple presence of their FA and the burden this brings. Also, a disease‐specific questionnaire can detect significant effects that general questionnaires could miss. The FASWLS will benefit researchers, clinicians, and patients, helping to resolve knowledge gaps and driving patient‐centered innovations in treatment, practice, and policy.

## AUTHOR CONTRIBUTIONS

**Gabriel Lins de Holanda Coelho**: conceptualization (lead); data curation (lead); formal analysis (lead); methodology (lead); writing – original draft (lead); writing – review and editing (supporting). **Melanie Lloyd**: writing – original draft (equal); writing – review and editing (equal). **Mimi L. K. Tang**: supervision (lead); writing – original draft (equal); writing – review and editing (equal). **Audrey DunnGalvin**: supervision (lead); writing – original draft (equal); writing – review and editing (equal).

## FUNDING INFORMATION

National Children's Research Centre, Grant/Award number: 2018 (C/18/12)

## CONFLICT OF INTEREST STATEMENT

The authors have no conflict of interest to declare.

## Data Availability

Derived data supporting the findings of this study are available from the corresponding author upon request.

## FA SATISFACTION WITH LIFE ITEMS

In most ways, my life with a food allergy is close to my ideal.

The conditions of my life with food allergy are excellent.

I am satisfied with my life with food allergy.

So far, I have all the important things I want in my life, despite my food allergy.

If I could live my life over, I would change almost nothing, including my food allergy.
